# Serum HDL-C subfractions as predictors of cardiovascular calcification in hemodialysis patients: novel insights and clinical implications

**DOI:** 10.3389/fmed.2024.1391057

**Published:** 2024-07-31

**Authors:** Dong-Yun Li, Wei Sun, Xiao-Tao Zhou, Yu Wen, Yang Zou

**Affiliations:** ^1^University of Electronic Science and Technology of China, Chengdu, China; ^2^Department of Nephrology, Sichuan Provincial Ziyang People’s Hospital, Ziyang, China; ^3^Hospital of Chengdu University of Traditional Chinese Medicine, Chengdu, Sichuan, China; ^4^Chengdu University of Traditional Chinese Medicine, Chengdu, Sichuan, China; ^5^Department of Nephrology, Sichuan Provincial People’s Hospital, School of Medicine, University of Electronic Science and Technology of China, Chengdu, Sichuan, China

**Keywords:** hemodialysis patients, cardiovascular calcification, high-density lipoprotein cholesterol, high-density lipoprotein3 cholesterol, cardiovascular

## Abstract

**Objective:**

This study aims to explore the relationship between cardiovascular calcification (CVC) and serum levels of high-density lipoprotein cholesterol (HDL-C) and its subfractions in hemodialysis (HD) patients.

**Methods:**

HD patients and healthy participants were recruited based on specific inclusion and exclusion criteria. Various blood indicators were measured, and demographic information was recorded. HDL-C particle levels were quantified using lipophilic fluorescent dye staining and capillary electrophoresis (microfluidic platform). Coronary artery calcium scores and valve calcification were used to classify HD patients into calcification and non-calcification groups.

**Results:**

Compared to healthy participants, HD patients showed a significant increase in HDL-C, high-density lipoprotein 2 cholesterol (HDL2-C), and high-density lipoprotein 3 cholesterol (HDL3-C) levels (*p* < 0.001). Further division of HD patients into calcification and non-calcification groups revealed higher serum HDL3-C concentrations (*p* = 0.002) and a higher HDL3-C/HDL-C ratio (*p* = 0.04) in the calcification group. Additionally, elevated HDL3-C levels were found to be an independent risk factor for CVC in HD patients (*p* = 0.040). The ROC curve analysis showed an AUC value of 0.706 for HDL3-C (*p* = 0.002).

**Conclusion:**

Our study indicates that elevated serum HDL3-C levels in HD patients are an independent risk factor for CVC and can serve as a potential predictor for CVC events. However, more studies need to verify its potential as a predictive indicator..

## 1 Introduction

End-stage kidney disease (ESKD) refers to the end stage of various chronic kidney diseases. According to the results of the 2019 Global Kidney Health Atlas of the International Society of Nephrology (ISN) involving 160 countries, the number of new diagnoses of ESKD worldwide reached 144 per million people (1). Currently, the incidence rate in European and Oceania countries is low (2). At the same time, the situation in North America (the main data comes from the United States) (3) and East Asia (China, Taiwan, Japan, and South Korea) is not optimistic (4, 5). Therefore, the disease has become one of the important challenges facing the global public health system.

High mortality is a major problem faced by ESKD patients (3, 6). The 5-year survival rate of patients receiving dialysis treatment is only 49% (7), and cardiovascular disease (CVD) is one of the main causes of death (8). Studies in various countries have reported that the mortality rate of ESKD patients due to CVD is approximately: 40% in the United States, 39.9% in Canada, 32.7% in Japan, 34.69% in Taiwan, China (9–12), and approximately 20% ~ 48.5% in mainland China (13, 14). Cardiovascular calcification (CVC) is an important cause of CVD and mortality in patients with ESKD (15). There are many causes of CVC, among which dyslipidemia is one of the key factors. It is generally recognized that lipid deposition can induce endothelial cell damage/death, chronic inflammation and local immune cell aggregation (especially macrophage infiltration), and ultimately promote calcification formation (16). Studies have shown that ESKD patients receiving hemodialysis have elevated triglycerides and decreased HDL-C levels (17). It is generally believed that higher HDL-C levels are closely associated with reduced adverse cardiovascular events, while conversely they increase the risk of CKD progression (18, 19). However, recent studies have yielded some new findings: (1) increased serum HDL-C concentration is negatively correlated with eGFR and even has a higher risk of cardiovascular and all-cause mortality (20, 21); (2) there were significant variations in HDL-C in ESKD patients (22). These new findings suggest that the impact of HDL-C changes on cardiovascular events in HD patients (ESKD stage) needs to be re-evaluated, especially the relationship between HDL-C subfractions and CVC. Therefore, we adopted a cross-sectional analysis research method to explore the potential relationship between changes in HDL-C subfractions and CVC in the hemodialysis population.

## 2 Materials and methods

### 2.1 Study design and participants

All participants who voluntarily enrolled in this study were recruited from the Ziyang People’s Hospital outpatient clinic in Sichuan Province between June 2019 and January 2020. In order to minimize potential confounding effects on lipid and general biochemical parameters, participants underwent thorough screening based on specific inclusion and exclusion criteria. The inclusion criteria comprised: ① Patients with ESKD undergoing regular maintenance hemodialysis (MHD) treatment for a minimum of 90 days; ② Individuals who provided informed consent to participate in the study. Conversely, the exclusion criteria encompassed: ① Patients below the age of 18; ② Individuals with a duration of MHD treatment of less than 90 days; ③ Patients employing peritoneal dialysis; ④ Patients diagnosed with active tumors; ⑤ Patients displaying acute inflammatory conditions; ⑥ Patients afflicted with severe liver-related disorders; ⑦ Pregnant individuals; ⑧ Individuals were unable to attend CT examinations effectively. Healthy controls were recruited on a voluntary basis. All study participants provided explicit written informed consent. All study participants provided explicit written informed consent. The study was conducted adhering to the ethical principles outlined in the Declaration of Helsinki. The Ethics Committees of Ziyang People’s Hospital granted approval for the study protocol (protocol code: No. 196 of 2019).

### 2.2 Demographic details

Demographic information, medical history, concurrent medication, current parameters related to dialysis treatment, CVD occurrences, duration of HD, and other pertinent details were systematically documented upon participants’ enrollment.

### 2.3 Standard serum biochemical analyses

All blood samples were exclusively collected from HD participants during mid-week dialysis sessions while in a fasting state (note: Blood samples were collected prior to dialysis treatment). The biochemistry measurements were performed utilizing commercially available kits and an autoanalyzer (Hitachi 7600–210, Hitachi Ltd., Tokyo, Japan). The measured parameters encompassed serum creatinine (Cr), blood urea nitrogen (BUN), uric acid (UA), cholesterol (CHOL), triglycerides (TG), low-density lipoprotein cholesterol (LDL-C), glucose (GLU), potassium (K), sodium (Na), calcium (Ca), phosphorus (P), albumin (Alb), alanine aminotransferase (ALT), aspartate aminotransferase (AST), and alkaline phosphatase (ALP).

### 2.4 Lipid measurements

The profiling of HDL-C subclasses was accomplished through capillary electrophoresis utilizing a microfluidics instrument (MICEP-30; Ardent BioMed, Guangzhou, China). Specifically, a separation matrix consisting of a linear-polymer solution of poly (N, N-dimethyl acrylamide) (Polysciences, Warrington, PA, United States) was employed. Serum samples, calibration materials, and quality-control materials were subjected to a 1:50 dilution in sample buffer (250 mM TAPS, pH 7.5), wherein a lipophilic fluorescent dye (Dyomics, Jena, Germany) was introduced, with an incubation period of 5–15 min preceding their loading onto the microfluidic chip’s wells. The chip run was initiated, executing a software script to apply predetermined currents and voltages in a systematic manner for separation. Laser-induced fluorescence at 680 nm facilitated the detection of lipoproteins stained with fluorescence. The entire protocol was completed within a one-hour timeframe. In addition, the statistical software integrated into the program autonomously computed the HDL2-C and HDL3-C subclasses in accordance with established algorithms.

### 2.5 Measurement of coronary artery calcium and heart valve calcification

Coronary artery calcification (CAC) Assessment: A non-contrast tomography cardiac tomography scan (256-slice CT, Siemens, Germany) was administered to all participants to acquire CAC data. The imaging was conducted using parameters of 120 kV, with mA tailored to individual body mass indices, and subsequently reconstructed through standard filtered back projection. The acquired images were subjected to analysis within a designated workstation (Brilliance Workspace), where the Agatston score was employed to quantify CAC levels. This reflects the CVC status of the subject.

Heart Valve Calcification Assessment: The presence or absence of atherosclerotic plaque in the carotid arteries was determined through ultrasound examinations conducted on all participants. The evaluation of heart valve calcification was executed using two-dimensional echocardiography. The extent of calcification at each valve was semi-quantified as being either absent or present. The same cardiologist evaluated all ultrasound images after the end of the dialysis session.

### 2.6 Statistical analyses

All statistical analyses were conducted using SPSS version 22.0 (SPSS Inc., Chicago, IL, United States). Continuous variables were represented as means ± standard deviation (SD) or medians with interquartile ranges, while categorical variables were presented as frequencies with corresponding percentages. The student’s t-test was employed to compare continuous variables, whereas the chi-square test was utilized for categorical variables. Subsequently, covariates that demonstrated statistical significance in the univariate analysis were selected for inclusion in the multivariate logistic regression analysis model. To further enhance the diagnostic assessment of selected variables, receiver operating characteristic (ROC) curve analysis was conducted. To ensure maximum statistical power, data missing ≤1% were included. All statistical tests were performed using two-sided *p* values, and statistical significance was indicated by a significance level of *p* < 0.05. GraphPad Prism 8.4.3 software (GraphPad Software, San Diego, California, United States) was used for graphing after statistical analysis.

## 3 Results

### 3.1 Comparison of serum HDL-C subclasses between healthy group and HD group

**Figure 1:**
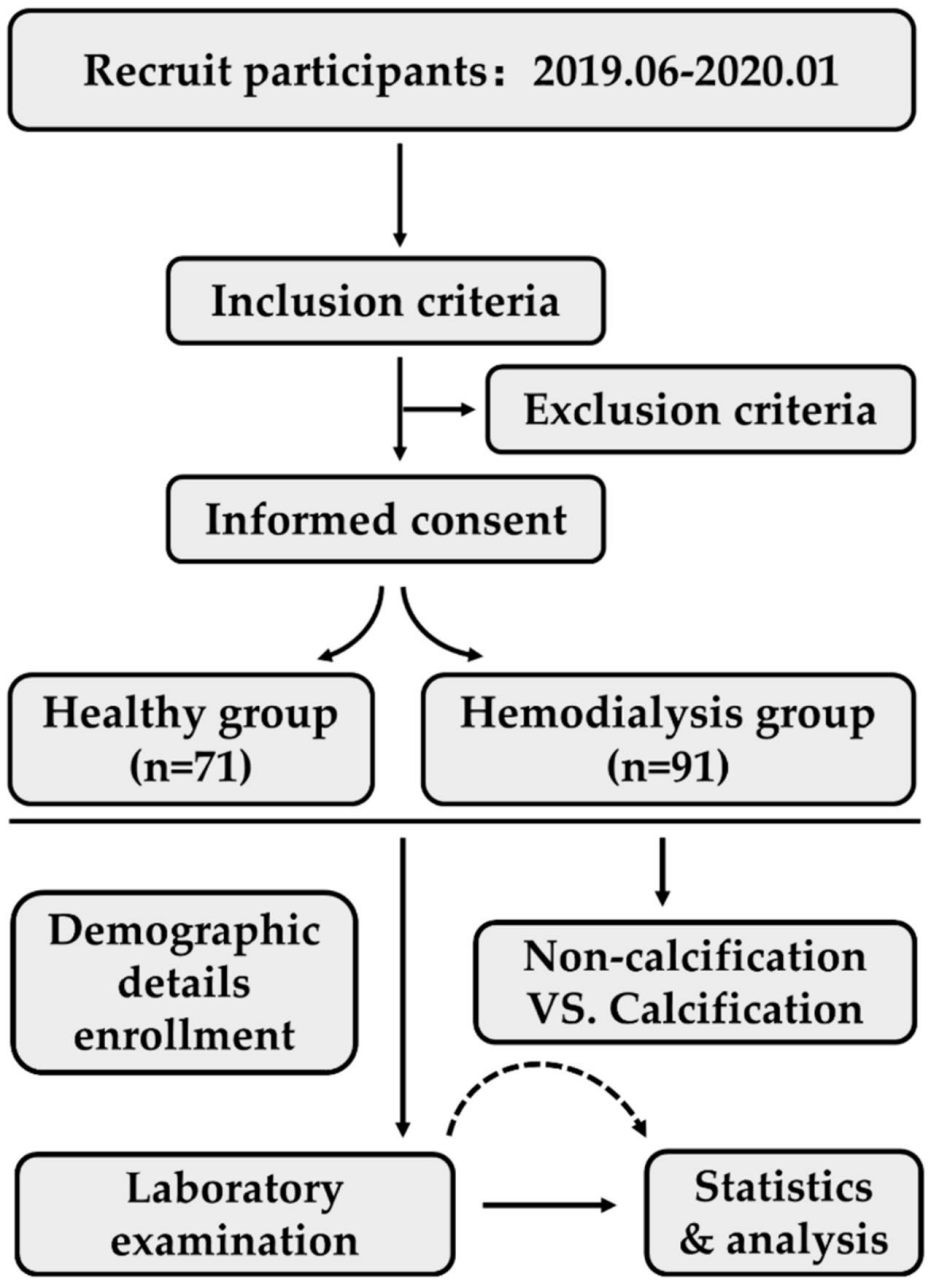
Flow chart of this study.

This study (The flow chart of this study is shown in Figure 1) comprised a total of 90 HD patients (male/female = 57/33) and 71 healthy participants (male/female = 35/36). The Age was 57.61 ± 13.35 years for HD patients and 54.92 ± 10.02 years for healthy participants. There was no statistical difference in the baseline values of age and gender between the two groups (gender, *p* = 0.540; age, *p* = 0.159). First, we examined serum creatinine in HD patients and healthy participants to confirm the status of renal function in the study population (*p* < 0.0001). Comparatively, HD patients exhibited significantly lower of serum HDL-C concentrations (1.01 ± 0.27 vs. 1.50 ± 0.33, *p* < 0.0001), serum HDL2-C concentrations (0.69 ± 0.21 vs. 1.03 ± 0.29, *p* < 0.0001), and serum HDL3-C concentrations (0.33 ± 0.17 vs. 0.47 ± 0.13, *p* < 0.0001) in contrast to healthy participants. Although serum concentrations of HDL-C and its subclasses were significantly lower in HD patients compared with healthy controls, however, there was no significant difference in the ratio of HDL-2C/HDL-C (67.97 ± 11.24 vs. 68.31 ± 6.99, *p* = 0.882) or HDL-3C/HDL-C (32.03 ± 11.24 vs. 31.69 ± 6.99, *p* = 0.882) between the two groups (data are shown in Table 1).

**Table 1:** Demographic characteristic and general biochemical parameters in hemodialysis patients and control subjects.

	Control group (*N* = 71)	HD group (*N* = 90)	t/chi square value	*p*-value
Sex (Male/Female)	35/36	57/33	0.375	0.540
Age (years)	54.92 ± 10.02	57.61 ± 13.35	−1.415	0.159
Cr (μmol/L)	71.69 ± 8.73	880.63 ± 291.91	−23.327	0.000*
HDL-C (mmol/L)	1.50 ± 0.33	1.01 ± 0.27	10.246	0.000*
HDL2-C (mmol/L)	1.03 ± 0.29	0.69 ± 0.21	8.681	0.000*
HDL3-C (mmol/L)	0.47 ± 0.13	0.33 ± 0.17	5.693	0.000*
HDL2-C/HDL-C (%)	68.31 ± 6.99	67.97 ± 11.24	0.225	0.822
HDL3-C/HDL-C (%)	31.69 ± 6.99	32.03 ± 11.24	−0.225	0.822

Cr, Serum creatinine; HDL-C, High-density lipoprotein cholesterol; HDL2-C, High-density lipoprotein2 cholesterol; HDL3-C, High-density lipoprotein3 cholesterol. *p < 0.05.

### 3.2 Comparison calcification of coronary artery or heart valves between calcification group and non-calcification group in HD patients

The HD patients were stratified into two distinct groups: the Calcification group, which exhibited calcification of the coronary artery or heart valves, and the non-calcification group. Table 2 presents the outcomes of univariate analysis concerning demographic characteristics and general biochemical parameters within the calcification and non-calcification groups.

**Table 2:** Demographic characteristic and general biochemical parameters in calcification and non-calcification group.

	Non-calcification (*n* = 28)	Calcification (*n* = 62)	t/chi square value	*p*-value
Age (years)	49.68 ± 13.14	61.19 ± 11.91	−4.112	0.000*
Sex (Male/Female)	11/17	29/33	0.438	0.647
BMI (kg/m^2^)	22.22 ± 3.15	24.15 ± 3.86	0.682	0.023*
Dialysis duration (month)	44.57 ± 39.47	55.64 ± 47.77	0.637	0.288
Hypertension (positive/total)	1/27	3/58	0.081	1.000
Diabetes mellitus (positive/total)	1/25	28/32	14.88	0.000*
Smoking (yes/no)	6/21	12/49	0.075	1.000
HDL-C (mmol/L)	0.95 ± 0.27	1.04 ± 0.27	−1.474	0.144
HDL2-C (mmol/L)	0.71 ± 0.26	0.68 ± 0.18	0.623	0.535
HDL3-C (mmol/L)	0.25 ± 0.06	0.36 ± 0.19	−3.166	0.002*
HDL2-C/HDL-C (%)	72.93 ± 8.19	65.73 ± 11.76	2.932	0.004*
HDL3-C/HDL-C (%)	27.07 ± 8.19	34.27 ± 11.76	−2.932	0.004*
HDL2-C/HDL3-C	3.08 ± 1.40	2.27 ± 1.10	2.954	0.004*
Cr (μmol/L)	908.53 ± 306.48	867.83 ± 286.67	0.609	0.544
BUN (mmol/L)	20.21 ± 4.62	21.78 ± 6.96	−1.086	0.281
UA (μmol/L)	430.11 ± 92.97	416.95 ± 101.59	0.583	0.562
CHOL (mmol/L)	3.64 ± 0.91	3.85 ± 1.23	−0.814	0.418
TG (mmol/L)	1.79 ± 1.20	1.96 ± 1.43	−0.556	0.580
LDL-C (mmol/L)	1.93 ± 0.65	2.02 ± 0.93	−0.456	0.650
PTH (pg/mL)	444.06 ± 362.18	461.11 ± 582.82	−0.169	0.866
GLU (mmol/L)	7.82 ± 3.32	10.67 ± 7.33	−1.967	0.052
K (mmol/L)	4.49 ± 0.79	4.69 ± 0.95	−0.990	0.325
Na (mmol/L)	139.58 ± 2.04	137.26 ± 3.85	2.989	0.004*
Ca (mmol/L)	2.19 ± 0.18	2.22 ± 0.24	−0.592	0.555
P (mmol/L)	1.84 ± 0.54	1.92 ± 0.75	−0.531	0.597
Alb (g/L)	40.78 ± 2.20	38.85 ± 3.58	2.628	0.010*
ALT (U/L)	19.20 ± 23.71	12.13 ± 9.08	1.529	0.137
AST (U/L)	21.18 ± 9.01	20.00 ± 9.10	0.573	0.569
ALP (U/L)	90.66 ± 31.00	104.51 ± 55.33	−1.235	0.220
Using calcium or active vitamin D (positive/total)	8/20	21/41	0.248	0.638

HDL-C, High-density lipoprotein cholesterol; HDL2-C, High-density lipoprotein2 cholesterol; HDL3-C, High-density lipoprotein3 cholesterol; Cr, Serum creatinine; BUN, Blood urea nitrogen; UA, Uric acid; CHOL, Cholesterol; TG, Triglycerides; LDL-C, Low-Density lipoprotein cholesterol; PTH, Parathyroid hormone; Glucose; K, Potassium; Na, Sodium; Ca, Calcium; P, Phosphorus; Alb, Albumin; ALT, Alanine transaminase; AST, Aspartate aminotransferase; ALP, Alkaline phosphatase. *p < 0.05.

Noteworthy differences emerged across several variables (the data in this section are all comparisons between the calcification group and the non-calcification group), including age (61.19 ± 11.91 vs. 49.68 ± 13.14, *p* < 0.0001), body mass index (BMI) (24.15 ± 3.86 vs. 22.22 ± 3.15, *p* = 0.023), the ratio of diabetic patients to total population in the group (28/32 vs. 1/25, *p* < 0.0001), proportions of HDL2-C/HDL-C (65.73 ± 11.76 vs. 72.93 ± 8.19, *p* = 0.004) and HDL3-C/HDL-C (34.27 ± 11.76 vs. 27.07 ± 8.19, *p* = 0.004), serum HDL-3C concentrations (0.36 ± 0.19 vs. 0.25 ± 0.06, *p* = 0.002), the ratio of HDL2-C/HDL3-C (2.27 ± 1.10 vs. 3.08 ± 1.40, *p* = 0.004), Na + levels (137.26 ± 3.85 vs. 139.58 ± 2.04, *p* = 0.004), and albumin levels (38.85 ± 3.58 vs. 40.78 ± 2.20, *p* = 0.010). While the serum HDL-C concentrations was slightly higher in the calcification group (1.04 ± 0.27 vs. 0.95 ± 0.27, *p* = 0.144), and the serum HDL2-C concentrations (0.68 ± 0.18 vs. 0.71 ± 0.26, *p* = 0.623) level was lower, these differences were not statistically significant.

### 3.3 Regression analysis

**Figure 2:**
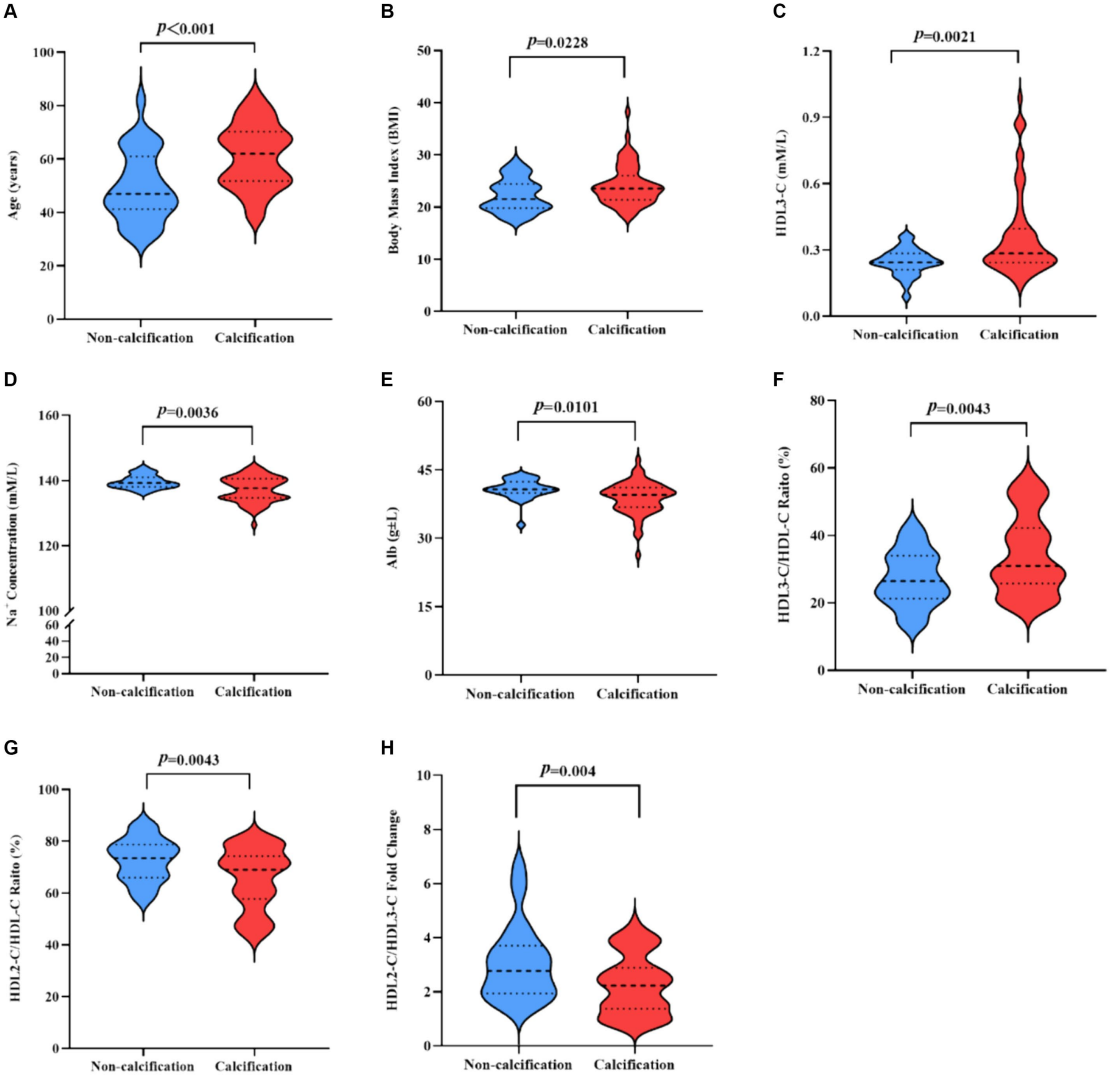
**(A,B)** Indicators of statistically significant differences between the calcification group and the non-calcification group in HD patients. T-test statistics were used. **(A)** Age. **(B)** BMI. **(C)** HDL3-C. **(D)** Na^+^ Concentration. **(E)** Abl. **(F)** HDL3-C/HDL-C ratio. **(G)** HDL2-C/HDL-C ratio. **(H)** HDL2-C/HDL3-C fold change. BMI, Body mass index; HDL3-C, High-density lipoprotein3 cholesterol; Alb, Albumin; HDL-C, High-density lipoprotein cholesterol; Na, Sodium; HDL2-C, High-density lipoprotein2 cholesterol.

The parameters exhibiting statistically significant differences (parameters are shown in Figure 2) were selected for subsequent multiple-factor logistic regression analysis analyses. The outcomes are presented in Table 3. We observed that HD patients with higher HDL3-C concentration (OR = 1.412, *p =* 0.040), advanced age (OR = 1.073, *p* = 0.009) and lower albumin levels (OR = 0.764, *p* = 0.021) were associated with a higher incidence of cardiovascular calcification. Therefore, low concentrations of Alb and HDL3-C and advanced age were identified as independent risk factors for CVC in HD patients.

**Table 3:** Multivariate logistic regression analysis for Calcification-HD patients.

Variable	OR	95%CI	*p*-value
Age	1.073	1.018–1.131	0.009*
HDL3-C	1.412	1.015–1.963	0.040*
Alb	0.764	0.609–0.960	0.021*

HDL3-C, High-density lipoprotein3 cholesterol; Alb, Albumin. *p < 0.05.

### 3.4 ROC curve analysis

**Figure 3:**
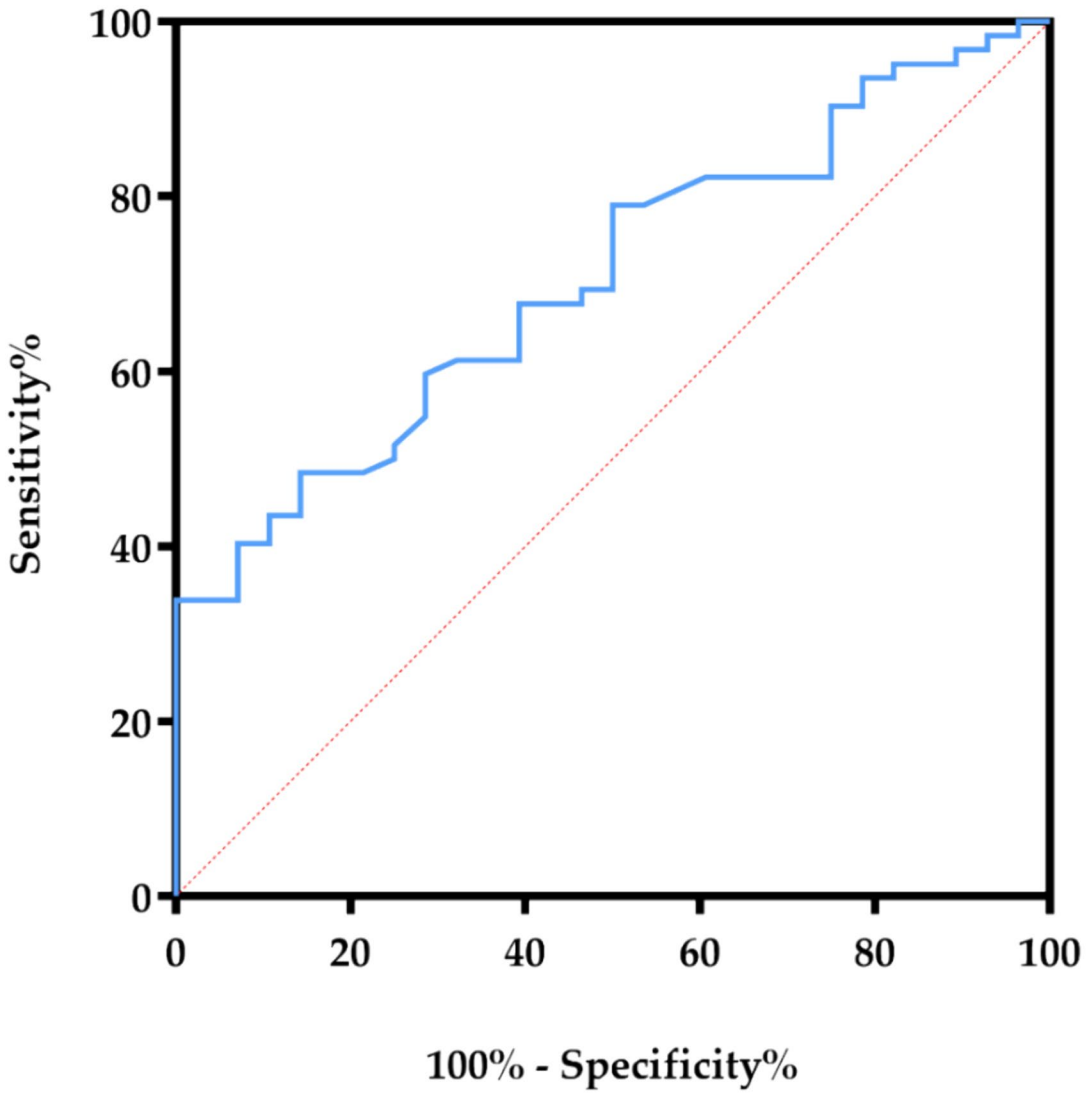
The ROC curves showed the correlation of HDL3-C concentration in predicting the specificity and sensitivity of CVC in HD patients. HDL3-C, High-density lipoprotein3 cholesterol.

Furthermore, we performed ROC curve analysis to evaluate the effect of the prediction of HDL3-C. The AUC value of the ROC curve of HDL3-C was 0.706 (*p* = 0.002). This suggests that HDL3-C may has the potential value in predicting CVC (the ROC curve graph is shown in Figure 3).

## 4 Discussion

HDL-C, a complex particle comprised of lipids and proteins facilitating cholesterol transport, exerts atheroprotective effects primarily through reverse cholesterol transport mechanisms. Consequently, it is implicated in mitigating the risk of CVD within the general population. However, despite its role in atheroprotection, the causative relationship between HDL-C and cardiovascular calcification remains enigmatic, particularly among HD patients. Previous studies have shown that HD patients have lower HDL-C concentrations than healthy people (23), and our results are consistent with this. However, when HD patients were comprehensively evaluated and classified using the two indicators of coronary artery calcification and heart valve calcification, we found that serum HDL-C levels may not be the key factor leading to CVC in patients. Another study had similar results to ours (24). In addition, we also found that the serum HDL-C subfractions concentrations in HD patients changed significantly compared with healthy volunteers, and the levels of serum HDL2-C and serum HDL3-C were significantly reduced. Therefore, we believe that for HD patients, it is the changes in HDL-C subfractions that are closely related to CVC, rather than serum HDL-C levels.

HDL-C is generally composed of two distinct subspecies, namely HDL2-C and HDL3-C. These subfractions differ not only in size but also in terms of unique biochemical, metabolic functions, and physiological characteristics. Past studies have suggested that HDL2-C does not play an important role in the process of CVC or CVD (25). Similar findings were found in our study. There was no statistically significant difference in HDL2-C concentration between calcified and non-calcified HD patients. This suggests that HDL3-C may be the main HDL-C subfraction that causes CVC in HD patients.

HDL3-C has traditionally been regarded as a protective factor against CVD (26). In addition, the reduction of HDL3-C is also considered to be related to the progression of CVD and is a potential predictor. Our findings confirmed that HDL3-C concentrations were reduced in HD patients compared with healthy volunteers. Interestingly, however, the situation was reversed when we further grouped the HD patients according to the presence or absence of coronary artery calcification and conducted statistical analysis. We found that HDL3-C concentrations were significantly higher and HDL2-C/HDL3-C ratios were lower in HD patients compared with patients without CVC. These results seem to indicate that HDL3-C has specific roles in different diseases. This also suggests that researchers and clinicians need to further explore and distinguish the role of HDL3-C in different disease states in the future.

Furthermore, we were curious why higher serum HDL3-C levels promote the course of CVC in HD patients. After literature search and combing, we think it may be related to the following two aspects. First, it is difficult to convert HDL3-C to HDL2-C. Under healthy conditions, phospholipid-cholesterol acyl transferase (LCAT) can promote the conversion of HDL3-C to mature HDL2-C (27), but related studies have shown that LCAT activity in HD patients is significantly reduced (28). It will lead to a decrease in HDL2-C levels and an increase in HDL3-C levels (29). In addition, HD patients have high levels of advanced oxidation protein products (AOPP) in their plasma (30). This substance blocks the binding of HDL to SR-B1, which results in reduced uptake of cholesteryl esters, which also reduces the conversion of HDL-2C to HDL3-C (31). Secondly, HDL3-C function is abnormal. Studies in healthy people show that HDL3-C has stronger antioxidant capabilities. This antioxidant effect is mainly related to paraoxonase 1 (PON1). PON1 is a lipid-dependent enzyme that relies on apoA-I to exert antioxidant effects. However, HDL3-C in ESKD patients has the ability to methylate apoA-I, thereby destroying the antioxidant capacity of PON1 (8). Therefore, the higher the HDL3-C level in HD patients, the more difficult it is for them to exert their normal antioxidant function.

CVD remains a significant concern for individuals undergoing HD and those with ESKD. A plethora of studies have consistently demonstrated that CVD stands as the foremost cause of comorbidity and mortality in ESKD patients (8). Thus, improving the predictive accuracy of CVC holds promise for enhancing the survival prospects of HD and ESKD patients. A previous investigation showcased the capacity of HDL3 to serve as a predictor of coronary artery atherosclerotic disease (CAAD) within a case–control cohort encompassing 1,725 participants (25).

Recent studies have suggested that HDL3 can be used as a potential marker for predicting arterial disease in patients with chronic kidney disease (32). In addition, our study also found that serum sodium, BMI index, and HDL3-C/HDL-C percentage were statistically different between the calcification and non-calcification groups. Although the percentages of HDL-C and HDL2-C were also statistically different, since there was no statistical difference in the concentrations of the two in the calcified and non-calcified groups, we speculated that their contribution to the CVC of HD patients was small, so no longer Included in subsequent analysis. On this basis, in order to further clarify the impact of HDL3-C on CVC in HD patients. Logistic regression was used to analyze the association between elevated HDL3-C levels and increased CVC incidence. The results of this study indicate that older age and low alb concentration are risk factors for CVC in HD patients, which is similar to the results of other studies (33). Additionally, statistical analysis results indicate that HDL3-C can also independently act as a risk factor for CVC in HD patients. We further used the ROC method to evaluate the sensitivity and accuracy of HDL3-C in predicting CVC. The results indicate that HDL3-C has a positive value in predicting CVC in the HD patient population. Considering the convenience of blood testing, we believe that HDL3-C shows some potential in predicting CVC in HD patients.

**Figure 4:**
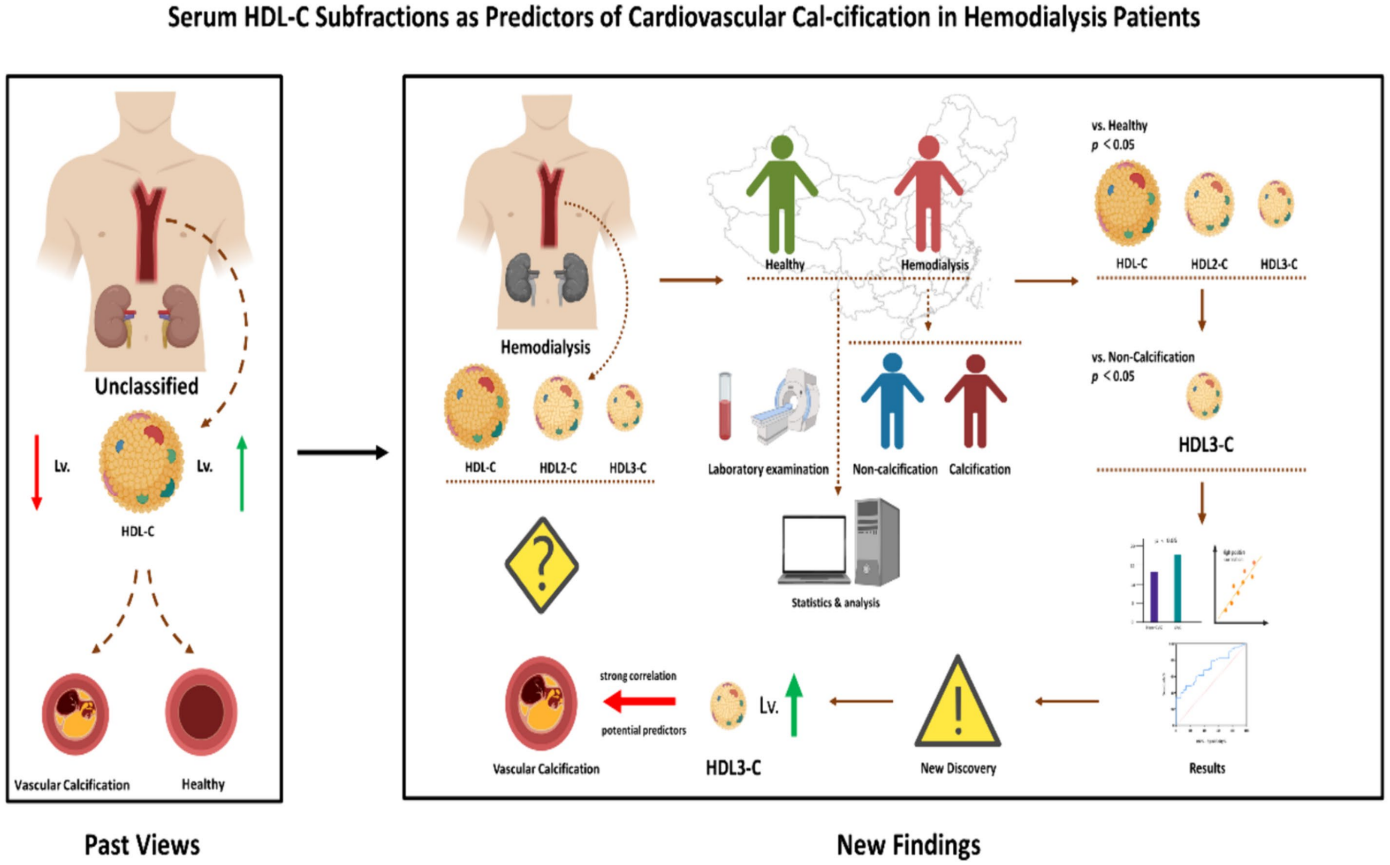
Different from the conventional situation described in past studies, we found that increased levels of HDL-C subfractions HDL3-C promote the progression of CVC in HD patients. HDL-C, High-density lipoprotein cholesterol; HDL3-C, High-density lipoprotein3 cholesterol; HDL2-C, High-density lipoprotein2 cholesterol; CVC, Cardiovascular calcification.

Several limitations of the current study must be considered. First, this study consisted entirely of participants from mainland China, limiting the inference of our data to participants from other regions or populations. Secondly, considering that the AUC value of serum HDL3-C did not reach 0.8, more clinical studies are needed in the future. Therefore, more clinical studies are needed in the future to finally determine its value in the clinical diagnosis of CVC. Third, the pathological role or mechanism of HDL3-C in CVC of HD patients is not completely clear. It must be emphasized that a clearer basic mechanism research is the cornerstone of its promotion value. Therefore, we hope that this study will also encourage more basic medical researchers to pay attention to the mechanism of HDL3-C in atherosclerosis and related diseases (Figure 4).

In summary, our study demonstrates abnormalities in HDL-C subclass composition in HD patients. Further analysis found that an increase in serum HDL3-C concentration, rather than a change in HDL2-C, was an important independent risk factor for CVC in HD patients. In addition, although HDL3-C showed the potential to predict CVC in HD patients, more clinical studies are needed to verify it.

## Data availability statement

The original contributions presented in the study are included in the article/supplementary material, further inquiries can be directed to the corresponding authors.

## Ethics statement

The studies involving humans were approved by the Ethics Committees of Ziyang People's Hospital granted approval for the study protocol (protocol code: No. 196 of 2019). The studies were conducted in accordance with the local legislation and institutional requirements. The participants provided their written informed consent to participate in this study.

## Author contributions

D-YL: Formal analysis, Writing – original draft, Writing – review & editing. WS: Formal analysis, Resources, Writing – review & editing. X-TZ: Writing – original draft, Writing – review & editing. YW: Supervision, Validation, Writing – review & editing. YZ: Data curation, Funding acquisition, Project administration, Supervision, Writing – original draft, Writing – review & editing.
